# Mid-Infrared Spectroscopy for Predicting Goat Milk Coagulation Properties

**DOI:** 10.3390/foods14132403

**Published:** 2025-07-07

**Authors:** Arianna Goi, Silvia Magro, Luigi Lanni, Carlo Boselli, Massimo De Marchi

**Affiliations:** 1 Department of Agronomy, Food, Natural Resources, Animals and Environment, University of Padova, Viale dell’Università 16, 35020 Legnaro, Italy; arianna.goi@unipd.it (A.G.); massimo.demarchi@unipd.it (M.D.M.); 2 Istituto Zooprofilattico Sperimentale del Lazio E Della Toscana “M. Aleandri”—National Reference Centre for Ovine and Caprine Milk and Dairy Products Quality, Via Appia Nuova 1411, 00178 Rome, Italy; luigi.lanni@izslt.it (L.L.); carlo.boselli@izslt.it (C.B.)

**Keywords:** mid-infrared spectroscopy, milk, goat, technological properties

## Abstract

The assessment of milk coagulation properties (MCPs) is crucial for enhancing goat cheese production and quality. In this study, 501 bulk goat milk samples were collected from various farms to evaluate the MCPs. Traditionally, cheesemaking aptitude is evaluated using lactodynamographic analysis, a reliable but time-consuming laboratory method. Mid-infrared spectroscopy (MIRS) offers a promising alternative for the large-scale prediction of goat milk’s technological traits. Reference MCP measurements were paired with mid-infrared spectra, and prediction models were developed using partial least squares regression, with accuracy evaluated through cross- and external validation. The ability of MIRS to classify milk samples by coagulation aptitude was evaluated using partial least squares discriminant analysis. Only the model for rennet coagulation time obtained sufficient accuracy to be applied for screening (R^2^_CrV_ = 0.68; R^2^_Ext_ = 0.66; RPD = 2.05). Lower performance was observed for curd-firming time (R^2^_CrV_ = 0.33; R^2^_Ext_ = 0.27; RPD = 1.42) and curd firmness (R^2^_CrV_ = 0.55; R^2^_Ext_ = 0.43; RPD = 1.35). Classification of high coagulation aptitude achieved balanced accuracy values of 0.81 (calibration) and 0.74 (validation). With further model refinement and larger calibration datasets, MIRS may become a resource for the dairy-goat sector to monitor and improve milk suitability for cheesemaking.

## 1. Introduction

The demand for milk and dairy products has consistently increased on a global scale, with a particularly notable rise in developing countries [1]. Although goat milk production represents a relatively small share of the global dairy market, following cow and buffalo milk [2], it is continuously growing.

The majority of goat milk is dedicated to cheese production, with most being consumed within the country of origin. The efficiency of cheese production and the quality of the final product largely depend on the compositional properties of the milk and its cheesemaking aptitude; that is, its ability to coagulate upon the addition of a clotting agent [3,4,5]. The parameters that define the coagulation process and the cheesemaking potential of milk are typically evaluated using lactodynamographic analysis, collectively referred to as milk coagulation properties (MCPs). These properties include (i) rennet coagulation time (RCT), which represents the interval (min) between the addition of the clotting agent and the onset of coagulation, (ii) curd-firming time (k_20_), indicating the time (min) needed to reach a curd firmness of 20 mm, and (iii) curd firmness (a_30_), defined as the coagulum firmness (mm) 30 min after the addition of the clotting agent [6]. Milk with good suitability for dairy processing is typically characterized by short RCT and k_20_ values, along with a relatively high a_30_ value. Generally, goat milk exhibits lower RCT and k_20_ values, and higher a_30_ values than cow milk [7]. This makes goat milk more advantageous for cheesemaking, as faster coagulation ensures more efficient processing, and rapidly reaching curd firmness indicates a firmer curd, which benefits both texture and yield [8,9]. However, goat milk shows a rapid decrease in curd firmness after reaching its maximum value [7].

Although the reference method provides reliable results, it remains impractical for large-scale milk recording programs. In fact, it requires considerable time, involves high costs, and depends on trained personnel to perform a labor-intensive analytical protocol [10]. To overcome these limitations, other approaches should be explored. Infrared spectroscopy, a rapid, cost-effective, and easily implementable technology widely used for food analysis worldwide, could serve as a potential alternative for this purpose. Specifically, mid-infrared spectroscopy (MIRS) exploits the vibrational absorption of infrared radiation by molecular bonds within matter in the spectral range of 2500–25000 nm (5000 to 900 cm^−1^), enabling the quantification of constituent concentrations. The accuracy of this technique is dependent on calibration models, sample conditions, and other contributing factors [11]. MIRS is particularly recognized in the dairy sector, as it is employed for the routine official analysis of liquid milk composition traits, including protein, casein, fat, lactose, and urea contents [12]. Given its widespread use, extending MIRS to assess coagulation properties would require minimal additional costs, offering an efficient and economical solution for large-scale screening. Moreover, studies have demonstrated its application in predicting the MCPs of individual bovine and ovine samples with sufficient or moderate accuracy [13,14], further supporting its potential for implementation in the dairy industry. By contrast, lower predictive accuracies have been reported in the literature for individual goat milk samples [15,16].

Despite the relevance of bulk milk in routine collection and processing, to the best of our knowledge, the use of MIRS to predict the coagulation properties of bulk goat milk has not yet been investigated. This lack of information limits our understanding of how this technique might perform under real-world conditions, where milk from multiple animals is pooled together. Addressing this gap is essential for assessing the practical applicability of MIRS in cheesemaking-oriented strategies and enhancing the technological valorization of goat milk.

The objectives of the present study were (1) to evaluate the accuracy of MIRS in predicting the MCPs of bulk milk samples from goats and (2) to assess the discriminant ability of spectra for the identification of samples with high coagulation aptitude.

## 2. Materials and Methods

### 2.1. Bulk Milk Sampling and Reference Standard Analysis

A total of 501 bulk milk samples (50 mL without preservative) were collected from 2021 to 2025 from goat farms located in central Italy, which reared different breeds, including Saanen, Alpine, Maltese, Murciano-Granadina, Sarda, Ciociara Grigia, and crossbreeds. The milking frequency on the farms ranged from once to twice per day, and some farms implemented out-of-season kidding practices to ensure year-round milk production. Bulk samples were collected from farm tanks, where milk from multiple goats was routinely pooled immediately after milking. Samples were obtained within the official milk quality system and transported to the laboratory under refrigeration at 4 °C. An analysis of samples was conducted within 36 h of collection at the quality milk laboratory of the Istituto Zooprofilattico Sperimentale del Lazio e della Toscana “M. Aleandri” (Rome, Italy). This laboratory is accredited by Accredia, the Italian Accreditation Body (Laboratory No. 0201A), and operates in compliance with the ISO/IEC 17025:2017 standards of the International Organization for Standardization. The chemical composition of milk (i.e., fat, protein, casein, and lactose content) was quantified using a MilkoScan^TM^ 7 RM (Foss Analytical A/S, Hillerød, Denmark) calibrated with the commercial goat standards obtained from the Milk Standard Laboratory of the Associazione Italiana Allevatori (Maccarese, Italy); the resulting spectrum of each sample, containing 1060 transmittance data points, was stored. The somatic cell count (SCC) was assessed using a Fossomatic™ FC system (Foss Analytical A/S, Hillerød, Denmark), and then converted to a somatic cell score using the formula of Ali and Shook [17]: SCS = 3 + log_2_ (SCC/100,000), while the MCPs (RCT, k_20_, and a_30_) were measured using a Formagraph LDG 2.0 (Ma.Pe System Srl, Firenze, Italy). For the coagulation trait analysis, sample preparation involved heating 10 mL of milk to 36 °C, followed by the addition of calf rennet (200 µL) composed of 75% chymosin and 25% pepsin (175 international milk clotting units/mL; Clerici S.p.A., Sacco srl, Cadorago, Italy) diluted to a 1.6% (*w*/*v*) concentration in distilled water. At the beginning of the analysis, an oscillating loop pendulum was placed in contact with the milk. As coagulation initiates, the viscosity of the milk increases, generating a resistance that is transmitted back to the loop. Consequently, the instrument produces a graphical representation of the curd firmness over time. Each measurement was performed within 30 min after adding the enzyme.

An index of milk aptitude to coagulate (IAC) standardized to mean = 100 and SD = 5 was built using the following formula [18]:

IAC = 100 + (a_30_ − mean_a30_)/sd_a30_ × 2·5 − (RCT − mean_RCT_)/SD_RCT_ × 2·5 (1)

where mean_a30_ and mean_RCT_ were the dataset averages of a_30_ and RCT, respectively, and SD_a30_ and SD_RCT_ were their standard deviations.

### 2.2. Development of the MIRS Prediction Models

**Figure 1:**
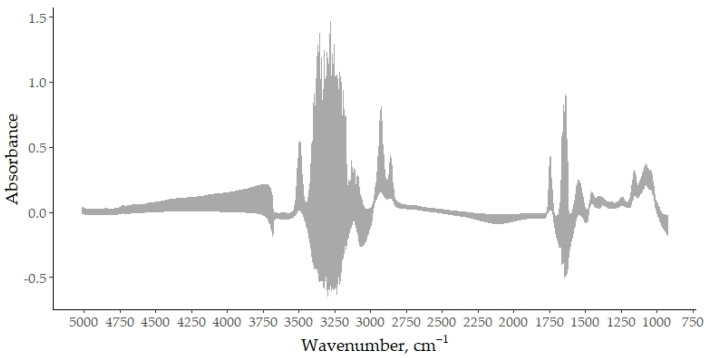
Raw mid-infrared absorbance spectra of bulk goat milk samples, recorded in the spectral range from 5000 to 900 cm^−1^, with a resolution of 1 cm^−1^ (1060 total wavenumbers).

As a first step in preparing the spectral data for analysis, the raw mid-infrared spectra of all goat bulk milk samples were transformed into absorbance data by taking the log_10_ of the reciprocal of the transmittance. This conversion allowed for the overall unprocessed spectral structure to be visualized and helped to identify the regions dominated by water absorption. Figure 1 shows the complete set of raw absorbance spectra prior to any exclusion or transformation, providing a comprehensive overview of the spectral variability within the dataset. In particular, the region between 3700 and 3000 cm^−1^ is characterized by broad and intense bands mainly attributed to the O–H stretching from water. The interval from 3000 to 2800 cm^−1^ includes bands related to the C–H stretching, primarily arising from lipids, as well as contributions from the region around 1800 and 1700 cm^−1^ [19]. The range between 1700 and 1600 cm^−1^ contains both the H–O–H bending vibration and the amide I band, mainly associated with the C=O stretching of the peptide bonds. The region from 1600 to 1200 includes the absorption bands corresponding to amide II and amide III, arising from protein and attributed to the C–N stretching vibrations in combination with the N–H bending, along with CH_2_ scissoring vibrations from lipid acyl chains. The range from 1200 to 900 cm^−1^ is dominated by the C–O stretching primarily associated to lactose and the P=O vibrations from phosphate-containing compounds [20]. This preliminary inspection of the spectra guided the subsequent removal of specific wavelength intervals that are known to be affected by water interference.

To minimize the inclusion of random noise, spectral regions related to water absorption (5011 to 2974 cm^−1^, 2503 to 1929 cm^−1^, 1712 to 1585 cm^−1^, and 964 to 925 cm^−1^) were excluded prior to the chemometric analysis in accordance with the standard practice reported by Grelet et al. [21]. To optimize the calibration accuracy, spectral outliers were first identified and removed based on the Mahalanobis distance (Global H > 3.0). The spectra were then coupled with the reference values of RCT, k_20_, and a_30_ to develop the respective prediction equations. Predictive equations for each MCP trait were built using modified partial least squares (mPLS) regression in the WinISI 4.10 software (Infrasoft International, Port Matilda, PA, USA). The mPLS regression analysis was followed by the elimination of chemical outliers setting a critical T-value of 3, removing samples whose predicted value deviated more than 3 standard errors of cross-validation from the reference value. Subsequently, a second mPLS was performed, followed by another round of outlier removal. Then, for each trait, samples were ordered by their reference values, and every fourth sample was assigned to the validation set, with the remaining 75% comprising the calibration set. The two subsets were ensured to exhibit similar means and SD for each target trait. Model calibration was thus performed on the calibration subset, internally tested through five-fold cross-validation, and externally validated on the reserved validation set. For the development of predictive models, spectral data were used either in their raw form or subjected to scatter correction techniques, including standard normal variate (SNV), detrending (D), a combination of SNV and D (SNV + D), and multiplicative scatter correction (MSC). These preprocessing techniques were further combined with the following mathematical treatments: 0,0,1,1; 1,4,4,1; 1,8,8,1; and 2,5,5,1 [22]. In these notations, the first digit represents the derivative order, the second indicates the gap over which the derivative is computed, the third specifies the number of data points used in the first smoothing, and the fourth corresponds to the number of data points in the second smoothing [22]. To prevent overfitting, the number of latent variables in the mPLS model was selected by minimizing the root mean square error computed at each cross-validation iteration. The optimal prediction equation was determined based on the coefficient of determination for cross-validation (R^2^_CrV_) and external validation (R^2^_Ext_), as well as the ratio of performance to deviation (RPD) during external validation. The variable importance in projection (VIP) scores from the final round of mPLS were retained for analysis. A *t*-test was performed using the TTEST procedure of SAS software v. 9.4 (SAS Institute Inc., Cary, NC, USA) to assess whether the bias, calculated as the average difference between the predicted and reference values, did not statistically differ from zero. The null hypothesis was tested at a significance level of 0.05, and *p*-values were used to determine the statistical significance.

### 2.3. Discriminant Analysis

The IAC variable was transformed into a binary trait using the mean value (100) as the threshold. Samples with IAC > 100 were classified as having a ‘high aptitude to coagulate’, while those with IAC ≤ 100 were classified as having a ‘low aptitude to coagulate’. A partial least squares discriminant analysis (PLS-DA) was performed using the ‘caret’ package [23] of R software v 4.4.2. to classify the milk spectra into these two categories. Model tuning was performed using 10-fold cross-validation repeated three times, and the number of components was set automatically but capped at a maximum of 15 to avoid overfitting. The spectral data points were mean-centered and scaled, and discrimination was performed based on class probabilities. The PLS-DA performance included sensitivity, specificity, positive predictive values, negative predictive values, balanced accuracy, and area under the curve (AUC) in both the calibration and validation. Balanced accuracy is the mean of sensitivity and specificity, and positive and negative predictive values are the proportions of positive and negative results that are true positive and true negative, respectively. For each metric, a 95% confidence interval was also reported.

## 3. Results and Discussion

### 3.1. Descriptive Statistics

The descriptive statistics of the MCPs and bulk milk chemical composition are shown in Table 1. The bulk milk samples used in the present study had fat, protein, and casein contents of 4.22%, 3.44%, and 2.64%, respectively. The average lactose content was 4.34%, whereas the average SCS was 6.77 (Table 1). The chemical composition of the evaluated milk samples was consistent with previous studies on bulk [8] and individual milk [16,24] of goats of the same breeds used in this study and reared in Italy.

Regarding the MCPs, in this study, the RCT and k_20_ averaged 9.59 min and 3.10 min, while the a_30_ averaged 29.78 mm (Table 1). A total of 8.98% of the samples did not coagulate during the analysis, and the lactodynamographic curve did not reach 20 mm. Consequently, for these samples, the k_20_ was considered missing data. Pazzola et al. [8] reported higher MCP values than those found in the present study, based on 432 bulk milk samples from 161 commercial goat farms with different breeds in Sardinia (Italy). Specifically, they reported RCT, k_20_, and a_30_ values of 13.20 min, 4.91 min, and 32.10 mm, respectively, in an extensive farming system, and 15.0 min, 4.50 min, and 32.80 mm, respectively, in an intensive farming system. Stocco et al. [16] also reported greater MCP values than those observed in the present study, based on 611 individual goat milk samples from different breeds (Alpine, Murciano-Granadina, Maltese, and Sarda) reared on 19 farms in Sardinia (Italy).

The chemical composition and the MCPs exhibited moderately high variability, likely due to the fact that the milk was bulk milk, originating from a multitude of farms and a diverse range of sampled breeds. In particular, the coefficient of variation (CV) for the MCPs ranged from 31% (RCT) to 44% (k_20_). A high degree of data variation is desirable when developing the MIRS prediction models for use in population-level phenotyping [15].

### 3.2. Predicting Milk Coagulation Properties Using MIRS

The prediction statistics of the models are presented in Table 2. The outliers identified and removed during model development were 6.0% for RCT, 7.5% for k_20_, and 7.2% for a_30_. The scatter correction applied was SNV combined with D for RCT, only SNV for k_20_, and D for a_30_, while the first derivatization order was used for each trait. The highest prediction accuracy was achieved for the RCT, whereas the least accurate model was for the k_20_, as confirmed by the RPD values of 2.05 and 1.35, respectively.

Under external validation, the models in the present study yielded R^2^_ExV_ values of 0.66, 0.27, and 0.43 for RCT, k_20_, and a_30_, respectively. Dadousis et al. [15] obtained lower R^2^ values for RCT and a_30_ (0.42 and 0.27, respectively) and a comparable value for k_20_ (0.29) in individual Sarda goat milk. In a multi-breed goat study, Stocco et al. [16] reported R^2^ values of 0.42, 0.47, and 0.48, showing better predictive accuracy for k_20_ and similar accuracy for a_30_, but still below the approximate screening benchmark of 0.66 proposed by Williams [25]. Given the limited literature on MIRS-based prediction of the MCPs in goats, parallelism can be drawn from the findings for other dairy species (i.e., cow, sheep, and buffalo). Moderate R^2^ values for RCT have also been described in ovine milk (0.59; [14]) and bovine milk (0.69; [13]). When considering the cross-validation approach, the R^2^_CrV_ value obtained for RCT in this study (0.68) was higher than that reported by Manuelian et al. [26] for individual buffalo milk (0.45), and under cross-validation was also higher than the value reported by Guerra et al. [27] for buffalo bulk milk (0.40), and by Boselli et al. [28] for sheep bulk milk. By contrast, the models developed for the k_20_ and a_30_ in the present study showed lower predictive ability than the RCT model, yielding R^2^_CrV_ values of 0.33 and 0.55, respectively (Table 2). These results are consistent with the cross-validation outcomes reported by Manuelian et al. [26], who obtained R^2^_CrV_ values of 0.39 for k_20_ and 0.51 for a_30_, and by Guerra et al. [27] for k_20_ (R^2^_CrV_ = 0.39). Meanwhile, higher R^2^_CrV_ values were reported for a_30_ by Guerra et al. ([27]; 0.61).

The differences in predictive accuracy may reflect methodological, technical, and biological factors. These include the frequency and quality of instrument calibration, size and representativeness of the calibration dataset, the choice and consistency of the spectral preprocessing methods, the chemometric approaches applied, and the rigor of both the calibration and the validation procedures [16]. Moreover, the biochemical expression of the target traits, the strength of their correlation with milk composition, and the extent to which relevant information is captured by mid-infrared spectral data should be considered. Fat, total protein, and casein fractions—quantified by MIRS—account for a sizable portion of bovine milk coagulation variability [6,7,29]. Studies on goat milk indicate that coagulation properties are less strongly associated with infrared-detectable components. In fact, in goats, breed effects are pronounced, and factors such as ionic calcium and pH contribute more significantly to coagulation behavior than the fat/protein ratio captured by MIRS [15,16,30].

**Figure 2:**
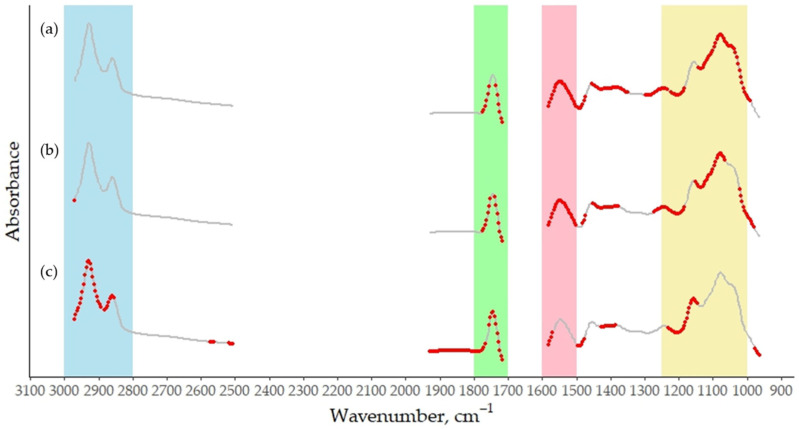
Average mid-infrared absorbance spectrum of bulk goat milk samples used for prediction of (**a**) RCT, (**b**) k_20_, and (**c**) a_30_, based on 338 selected wavenumbers (from an original set of 1060), after removing regions dominated by water absorption. Red dots indicate the most informative wavenumbers, while the remaining wavenumbers are shown in grey. Regions in blue and green are known as the milk region “Fat B” and “Fat A”, respectively [20]. The pink and yellow band represents the spectral window associated with milk protein and lactose [19].

The RPD value closest to 2, which is the commonly accepted threshold for a prediction model to be considered suitable for quantitative screening [31], was that of the RCT. From a methodological perspective, the higher predictive performance observed for the RCT may be partially attributed to its association with the initial enzymatic phase of milk coagulation, which is more directly and consistently reflected in the mid-infrared spectrum of milk. By contrast, traits related to the later stages of curd development involve both chemical and physiomechanical changes, making their spectral signatures in milk more indirect and overlapping, resulting in more complex information to predict [32]. Overall, the predictive performance of the k_20_ and a_30_ models remains insufficient to enable the reliable application of MIRS for quantifying these traits in a screening context. The R^2^ values in external validation, 0.27 and 0.43, respectively, and the RPD values, 1.42 and 1.35, respectively, fell below the thresholds generally considered acceptable for reliable phenotyping, reflecting poor predictive performance. In particular, the R^2^ results indicate that the models capture less than half—and in the case of the k_20_, less than one-third—of the variance in the reference data. Therefore, the application of the models for rough large-scale phenotyping or exploratory purposes is currently unfeasible. However, combining MIRS data with additional phenotypic or genomic information could, in the future, yield more reliable prediction models than those based on a single source of information [33]. Nonetheless, the use of these models in routine milk recording or precise decision-making remains premature without further refinement. The development of new trait-specific preprocessing techniques or the use of advanced machine learning algorithms may enhance the predictive capacity of the MIRS models for MCP traits, particularly for complex traits, such as k_20_ and a_30_. The most informative spectral regions for the MCP prediction were identified using the VIP scores (Figure 2), with wavenumbers exhibiting VIP values greater than 1 being considered the most significant [34]. As shown, every trait contributes—albeit to varying degrees—to the fat, protein, and lactose-related bands [19,20]. Notably, only a_30_ places particular weight on the “fat B” region (3000 to 2800 cm^−1^) [19], which corresponds to the C–H stretching of the methyl group in milk [35]. By contrast, for all the MCPs, high VIP scores were reported at wavenumbers in the “fat A” region (1800 to 1700 cm^−1^), corresponding to changes in the C=O stretching vibration of ester linkage in bovine milk [19].

### 3.3. Discriminating High Milk Coagulation Aptitude Using MIRS

**Figure 3:**
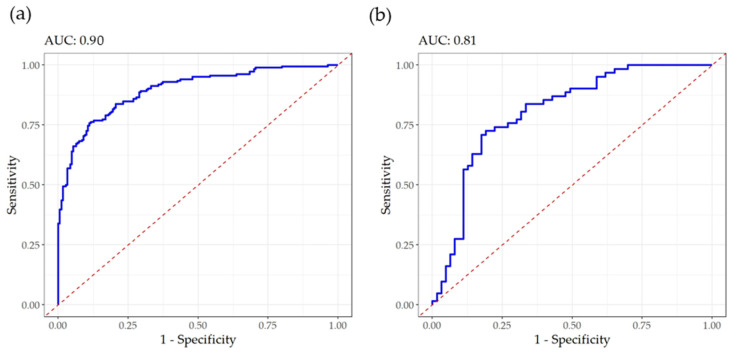
Receiver operating characteristic curves for goat bulk milk coagulation aptitude in calibration (**a**) and validation (**b**), with the corresponding area under the curve (AUC). The samples classification was ‘high aptitude to coagulate’ (IAC > 100) and ‘low aptitude to coagulate (IAC ≤ 100).

To assess the discriminant ability of the mid-infrared spectra in identifying the samples with high coagulation aptitude, a PLS-DA was performed. In the dataset, 50% of the milk samples had high aptitude to coagulate (IAC > 100) (Table 3). Sensitivity, defined as the probability of correctly identifying milk samples with a high aptitude to coagulate (IAC > 100), was 0.79 in the calibration and 0.68 in the validation (Table 3). Specificity, which is complementary to sensitivity, represents the probability of correctly identifying milk samples with low aptitude for coagulation (IAC ≤ 100). This parameter was 0.82 in the calibration and 0.81 in the validation. Martín-Gómez et al. [36] recommended an optimization based on sensitivity and specificity to assess the quality of the PLS-DA models that discriminate between the two classes. The balanced accuracy was 0.81 in the calibration and 0.74 in the validation. Moreover, the AUC values were 0.90 in calibration and 0.80 in validation (Figure 3), suggesting that the model has good discriminative ability during training and maintains an acceptable performance when applied to new data.

Considering that the predictive performance of the MCPs from the spectral data was moderate, binary phenotypes derived from the PLS-DA could serve as a valid indicator of traits for various applications, such as milk payment systems. Currently, in both the goat and cow milk industries, payment systems are primarily based on quantity and quality, with bonuses or penalties applied to farmers according to the fat and protein content as well as the SCC. Including a phenotype related to high or low coagulation aptitude could be a valuable tool for improving the suitability of goat milk for cheesemaking. This approach could incentivize the production of milk with better technological properties, thereby enhancing both the product quality and the economic returns for farmers.

### 3.4. Future Perspectives

Future research should aim to expand the calibration dataset by incorporating greater variability for factors such as lactation stage and farming system, which were not explicitly considered in the present study. The inclusion of multiple breeds already provided a degree of biological diversity, and further broadening of the management and production-related variables may help refine the model, enhancing its robustness and applicability across different contexts. As a future application, the development of such models with portable or handheld near-infrared and mid-infrared spectrometers—already explored for assessing milk composition, quality traits, and adulterants [37,38,39]—could open new opportunities for on-site screening of the MCPs, although their use to predict these parameters has not yet been established. Moreover, the prediction of the IAC, an index that incorporates both the RCT and a_30_, could be a valid tool for milk payment systems, helping to distinguish and reward farms producing milk with a higher aptitude to coagulate compared to those with a lower aptitude. However, to be effectively implemented in such systems, it is necessary to expand the dataset to capture greater variability and improve the robustness and the generalizability of the predictive models.

## 4. Conclusions

This study highlights the potential of MIRS as a tool for predicting the RCT in goat milk and assessing the discriminant ability of spectra to identify samples with high coagulation aptitude. Although the models developed still require improvements to achieve full reliability across all the traits included, the promising results for RCT prediction suggest that MIRS could serve as a valuable starting point for developing rapid and cost-effective screening methods. The other models (k_20_ and a_30_) require further refinement before practical application can be considered, including the incorporation of larger and more diverse datasets and the integration of a wider range of factors related to farm management and production conditions. Moreover, MIRS can be a reliable tool for classifying samples based on their coagulation aptitude (high or low). Beyond its analytical potential, this approach is relevant to the dairy industry, where fast and affordable methods for assessing milk quality are increasingly needed to support product standardization and valorization. Validated models with good accuracy could be implemented in milk collection centers or on farms to support rapid decision-making, and MIRS could gain the potential to become a resource for the dairy goat industry to monitor and enhance milk quality for cheesemaking and improve product consistency.

Future research should focus on refining the prediction models by expanding the calibration datasets to include a wider range of factors to fully exploit the potential of this technology in supporting quality-oriented strategies.

## Figures and Tables

**Table 1:** Descriptive statistics of milk coagulation traits and chemical composition for goat bulk milk.

Trait ^1^	N ^2^	Mean	SD	Range	CV (%)
Milk coagulation properties					
RCT, min	501	9.59	2.94	1.00–26.30	30.70
k_20_, min	456	3.10	1.74	0.52–11.15	44.30
a_30_, mm	501	29.78	11.96	2.90–62.62	40.16
IAC	501	100.00	3.48	84.06–113.06	3.28
Milk quality traits					
Fat, %	476	4.22	1.15	1.06–12.1	27.31
Protein, %	479	3.44	0.54	2.26–6.41	15.76
Casein, %	479	2.64	0.59	1.39–6.23	22.40
Lactose, %	479	4.34	0.31	3.37–6.04	7.14
SCS	479	6.77	1.34	0.88–11.07	19.79

^1^ RCT, rennet coagulation time; k_20_, curd-firming time; a_30_, curd firmness; IAC, index of milk aptitude to coagulate, defined as IAC = 100 + (a_30_ − mean_a30_)/sd_a30_ × 2·5 − (RCT − mean_RCT_)/SD_RCT_ × 2·5; SCS, somatic cell score. ^2^ N, number of samples prior to reference outliers’ elimination.

**Table 2:** Fitting statistics ^1^ of modified partial least square regression models using external validation for coagulation properties ^2^ of goat bulk milk with mid-infrared spectroscopy (MIRS).

Trait	Calibration Set (75%)					Validation Set (25%)				
	LF	SC	MT	R^2^_CrV_	SE_CrV_	Bias	Slope	R^2^_ExV_	SE_P_	RPD_ExV_
RCT, min	11	SNV + D	1,4,4,1	0.68	1.57	−0.12	0.87	0.66	1.58	2.05
k_20_, min	9	SNV	1,4,4,1	0.33	1.09	−0.01	0.76	0.27	1.13	1.42
a_30_, mm	8	D	1,4,4,1	0.55	7.90	0.24	0.91	0.43	9.03	1.35

^1^ LF, latent factors; SC, scatter correction; MT, mathematical treatment; R^2^_CrV_, coefficient of determination of cross-validation; SE_CrV_ = standard error of cross-validation; R^2^_ExV_ = coefficient of determination of external validation; SE_P_, standard error of external validation; RPD = ratio of prediction to deviation calculated as the ratio between the standard deviation of the observed trait and the SE_P_. ^2^ RCT, rennet coagulation time; k_20_, curd-firming time; a_30_, curd firmness at 30 min after rennet addition to milk.

**Table 3:** Performance with 95% confidence interval of partial least square discriminant analysis for the prediction of coagulate aptitude ^1^ of goat bulk milk using mid-infrared spectroscopy (MIRS).

Performance	Calibration Set (75%)	Validation Set (25%)
Prevalence	0.51	0.50
Sensitivity	0.79 (0.73–0.85)	0.68 (0.55–0.79)
Specificity	0.82 (0.76–0.87)	0.81 (0.69–0.90)
Positively Predictive Value	0.82 (0.76–0.87)	0.78 (0.65–0.88)
Negatively Predictive Value	0.80 (0.73–0.85)	0.71 (0.59–0.82)
Balanced Accuracy	0.81 (0.75–0.86)	0.74 (0.62–0.85)

^1^ Two classes: samples with ‘high aptitude to coagulate’ (IAC > 100) and ‘low aptitude to coagulate (IAC ≤ 100).

## Data Availability

The original contributions presented in this study are included in the article, further inquiries can be directed to the corresponding author.
